# Development of Geopolymers as Substitutes for Traditional Ceramics for Bricks with Chamotte and Biomass Bottom Ash

**DOI:** 10.3390/ma14010199

**Published:** 2021-01-04

**Authors:** Juan María Terrones-Saeta, Jorge Suárez-Macías, Francisco Javier Iglesias-Godino, Francisco Antonio Corpas-Iglesias

**Affiliations:** Department of Chemical, Environmental, and Materials Engineering, Higher Polytechnic School of Linares, University of Jaen, Scientific and Technological Campus of Linares, 23700 Linares, Jaen, Spain; jsuarez@ujaen.es (J.S.-M.); figodino@ujaen.es (F.J.I.-G.); facorpas@ujaen.es (F.A.C.-I.)

**Keywords:** geopolymer, chamotte, biomass bottom ash, ceramic, circular economy, environment

## Abstract

The greater environmental awareness, new environmental regulations and the optimization of resources make possible the development of sustainable materials as substitutes for the traditional materials used in construction. In this work, geopolymers were developed as substitutes to traditional ceramics for brick manufacture, using as raw materials: chamotte, as a source of aluminosilicate, and biomass bottom ashes from the combustion of almond shell and alpeorujo (by-product produced in the extraction of olive oil composed of solid parts of the olive and vegetable fats), as the alkaline activator. For the feasibility study, samples were made of all possible combinations of both residues from 100% chamotte to 100% biomass bottom ash. The tests carried out on these sample families were the usual physical tests for ceramic materials, notably the compression strength test, as well as colorimetric tests. The freezing test was also carried out to study the in-service behavior of the different sample groups. The families with acceptable results were subjected to Fourier transform infrared (FTIR) analysis. The results of the previous tests showed that the geopolymer was indeed created for the final families and that acceptable mechanical and aging properties were obtained according to European standards. Therefore, the possibility of creating geopolymers with chamotte and biomass bottom ashes as substitutes for conventional ceramics was confirmed, developing an economical, sustainable material, without major changes in equipment and of similar quality to those traditionally used for bricks.

## 1. Introduction

The construction sector is one of the most demanding sectors in terms raw materials and the one that causes the greatest greenhouse gas production [1,2]. This fact is mainly due to the high production of materials as well as their low cost. More specifically, the consumption of ceramic materials in the building sector, among others, causes the scarcity of natural resources such as clay [3,4,5] as well as generates significant CO_2_ emissions due to poorly optimized industrial processes [6]. Moreover, the construction sector accounts for the largest percentage of the global energy consumption [7].

On this basis, and with new circular economy trends, in recent years research lines have been developed based on the creation of construction materials with the incorporation of waste [8,9,10]. Thus, it is possible to reduce the extraction of virgin materials and to take advantage of waste from other industries [11,12,13,14]. Therefore, the economic and ecological flows of the materials are closed [15]. Moreover, in the field of ceramic materials, manufacturing processes produce high CO_2_ emissions mainly due to the high temperatures generated in their manufacture, around 950 °C for traditional ceramics.

It is therefore essential to search for new materials with more optimized manufacturing processes that use residues in their composition, have good qualities and that can serve at the end of their useful life for the creation of other materials [16]. Based on this, in recent years, different lines based on geopolymers have been developed as cement substitutes or [17,18,19,20], as in this research, geopolymers as substitutes for traditional ceramic materials such as construction bricks. The term geopolymer was coined in 1978 by Joseph Davidovits [21] and is one of the most promising materials for the construction sector [22,23].

The geopolymer is an inorganic polymer [24] formed by the reaction of a source of aluminosilicate (binder) with an alkaline solution (activator) [25]. In this process called geopolymerization, aluminate and silicate monomers are formed, then they become oligomers and finally geopolymers. The water in this process is depleted, so the drying conditions are very important for its end resistance [26]. 

The sources of aluminosilicates that have been used correspond mostly to waste. Among these wastes are coal fly ash [27,28,29,30], slag waste from metallurgical industries [31,32,33,34], metakaolin [35,36,37], glass wastes [38,39,40], bagasse [41,42,43] and even hazardous waste [44,45,46,47]. It is therefore a material that not only reduces the emissions of other materials such as cement [48] or the extraction of clay for ceramic materials, but also its manufacturing process emits less greenhouse gases [49,50] and uses waste from other industries as raw materials [51]. The geopolymer is therefore a green material for the environment [52,53,54] and it is framed within the new circular economy.

In turn, sodium hydroxide or potassium hydroxide in appropriate proportions have been used for activator or alkaline solutions. An increase in the concentration of both would cause the rapid precipitation of the aluminosilicate gel and lower its compressive strength [55]. In addition, a low concentration of activator would cause an incomplete geopolymerization process and lower compressive strength. Therefore, the concentration of the activator as well as the curing temperature [56] is essential for obtaining the best mechanical properties [57]. The properties of geopolymers are diverse, and include high temperature resistance [58,59,60], piezoelectric properties [61], and good behavior in contact with steel [62].

Based on what has been said and in order to develop substitutes for traditional ceramics that are less harmful to the environment [63], geopolymers with chamotte and biomass bottom ash were developed in this paper. The source of aluminosilicate is the chamotte and the activator is the biomass bottom ash, since the ashes have a high percentage of potash because they correspond to the combustion of almond husks and alpeorujo. It is therefore a material that uses waste as raw materials and more optimized manufacturing processes [64].

As mentioned, the building consumes huge amounts of virgin materials to obtain new materials [65]. The increasing construction of buildings and the renovation of existing ones cause more materials to be consumed and in turn to produce more waste [66]. Companies that manufacture bricks with red clay generate a large amount of waste, mainly, by bricks that do not have the right geometric and visual shapes or breaking in the transport [67]. These bricks are crushed to deposit them in the vicinity of the industries with the consequent environmental impact. This material derived from the manufacture of defective and crushed red clay bricks is called chamotte [68]. The chamotte has had a use in civil engineering as a filler material or in the ceramic industry itself as an additive for stoneware or new bricks [69,70], however, in these processes, their properties are not optimized. The composition of the chamotte with high proportions of silica and alumina make it ideal for use in geopolymers. Some authors have studied geopolymers with chamotte and sodium hydroxide as an activator [71,72,73], however, the literature is scarce and the development of timely studies in this area is still necessary.

On the other hand, the biomass bottom ash depends on their composition of the biomass used in combustion, so each case must be studied. This waste is a big problem since the global biomass production is 140 billion tons per year [74]. A lot of research is being carried out for biomass flying ashes [75] and very few studies for biomass bottom ashes.

Biomass bottom ashes are a residue with inorganic components and to a lesser extent with organic ones [76]. Its quality and composition depends on the biomass used and the combustion process [77], being characterized as a non-hazardous waste according to European legislation [78]. However, it is currently an environmental problem when produced in large quantities, not valorizing it and depositing it in landfill. Although, there are few studies on its valorization that have been successful [79,80,81,82].

Therefore, the scope of this research is to develop geopolymers with one hundred percent waste, chamotte and biomass bottom ash, as substitutes for traditional ceramics. For this, different groups of samples were made with different percentages of both materials, conformed with water after going through a drying process. Finally, its physical, compressive strength, and colorimetric properties were studied and its durability was evaluated with the freezing test traditionally used in ceramics. Samples manufactured with different percentages of waste combination that reflected suitable results in the previous tests were analyzed with Fourier transform infrared (FTIR).

In short, with the development of this new geopolymer material as a substitute for traditional ceramics for the manufacture of bricks, a series of obvious economic and environmental advantages are achieved. On the one hand, the cost of clay extraction is reduced, as well as the cost of the manufacturing process by preventing high-temperature sintering of the ceramics and in addition, the cost of waste is practically non-existent, as it is not used at present. From an environmental point of view, it can be said that the impact on the landscape and the environment is reduced, as well as greenhouse gas emissions, since it is not necessary to extract raw materials and avoid depositing the waste in dumps. In addition, the production process is optimized by reducing the emission of harmful gases without major modifications to the machinery used. Finally, a new life is given to the waste that is not currently used, closing the flow of materials and developing a new circular economy.

## 2. Materials and Methods 

The materials used in this work as well as the methodology followed are detailed in the following paragraphs.

### 2.1. Materials

The materials used are entirely industrial by-products. On the one hand, (and as a source of aluminosilicates) the commonly called chamotte will be used. Chamotte is a by-product derived from the ceramic industry that is the basis of the material for the shaping of the geopolymer after activation. In turn, the biomass bottom ash of almond husk and alpeorujo combustions will be used as an alkaline activator, henceforth biomass bottom ash (BBA).

Therefore, since both by-products will be analyzed in depth in the methodology, successive paragraphs shall describe their origin and training.

#### 2.1.1. Chamotte

Chamotte is an inherent industrial by-product of ceramics production. The samples taken belong to the companies of the province of Jaen, Spain. These companies are dedicated to the manufacture of bricks with red clay.

In the manufacturing process, bricks that are not accepted for commercialization are discarded mainly because of their dimensions or shapes. Given their volume, they are crushed in the plant to be able to store it more easily and if possible, its subsequent withdrawal for use in other activities, such as the filling of embankments, sports courts, etc.

Based on the above, the by-product used was almost entirely sintered according to an appropriate process, so it offers stable physical and chemical characteristics. Since the process is similar between the different brick manufacturing companies as well as the raw material used, there is a repeatability of the properties of the by-product over time.

This material after its process is easily found in very fine grading, so its use is immediate within the conformed of the geopolymer.

#### 2.1.2. Biomass Bottom Ash from the Combustion of Almond Husk and Alpeorujo

The biomass bottom ash used in this project, hereinafter BBA, belongs to the companies located in Jaen, Spain. These biomass bottom ashes correspond to the by-product generated in the combustion of the almond husk and alpeorujo for the generation of electrical energy.

Using such a specific combustion material creates a by-product of similar physical and chemical properties over time. This material will be analyzed in the following sections and has the fundamental role of providing the alkaline activation of the chamotte for the formation of the geopolymer, and consequently, its mechanical properties.

It should be noted that before using the by-product, it was crushed to obtain a fine grain size. The process of crushing the biomass bottom ash, which has a maximum particle size of 16 mm, was carried out with the same equipment used for crushing clay in the ceramics industry. Furthermore, as the biomass bottom ash has low resistance, due to the materials from which it comes, the process is fast, economical and of high quality.

### 2.2. Methodology

The methodology to be followed in this work is clear and objective to evaluate the possibility of geopolymer conformation through the use of by-products of the ceramic industry and the generation of energy. The main purpose is to create a sustainable and economical material as a substitute for traditional ceramics.

First of all, both by-products were analyzed in order to determine their chemical composition. In this way, the present elements and compounds, capable of fulfilling the functions of aluminosilicate and alkaline activator, were evaluated, respectively. The physical properties were evaluated to determine the ease of the material for its treatment and its subsequent conformation in the successive processes.

Once both by-products were analyzed, different samples with different combination percentages were formed. Taking as a base material the chamotte, increasing percentages of biomass bottom ash were added from 10% to 100%, with increases of 10%. In this way, the variation could be observed of the physical properties of the geopolymer in all possible combinations of both elements.

The two residues were mixed in the corresponding percentage and conformed in a matrix with a pressure of 30 ± 1 MPa. Once the samples were formed, their dimensions were measured and they were dried at room temperature (20 ± 2 °C) for 24 h and at 90 ± 2 °C for another 24 h.

After the drying process was carried out, we proceeded to leach the elements that had not reacted and involved a useless load. This phase consisted of a continuous recirculation of water in a tank after submerging the samples. Once this process was carried out in the laboratory, the samples were dried again at a temperature of 90 ± 2 °C for 24 h, finally measuring their dimensions and weight.

The physical tests after the conformed samples are the typical tests performed on the ceramic elements to confirm the quality. Moreover, the aesthetic properties of the test sample families and the compressive strength were studied. 

Finally, an accelerated ageing test was conducted to evaluate the behavior of different families over time. In this case, and because it is one of the most common tests used for ceramics, the freezing test was carried out. The result of this test was assessed visually.

In a final point, all the results obtained from the different families were analyzed to obtain a combination field of both residues that create a suitable geopolymer according to the European ceramics regulations. The combinations of chamotte and biomass bottom ash that showed acceptable results in the tests were finally analyzed with Fourier transform infrared (FTIR). In this way, the formation of the geopolymer in these combinations as well as the variations between them could be observed.

Based on the comments and according to the logical scheme, the following subsections will be divided into several groups, the initial tests of the by-products, the geopolymers conformed and ageing tests and Fourier transform infrared.

#### 2.2.1. Initial Tests of the by-Products

Based on the comments, and as an initial and essential premise of this work, the industrial by-products, chamotte and biomass bottom ash were analyzed in detail.

First, both by-products were crushed and sieved by the 0.25 mm sieve and then dried at a temperature of 105 ± 2 °C. The resulting material was the one that had been used in all the tests of this work and in the conform of geopolymers.

It should be taken into account that the humidity of the products under study for the conformation of the geopolymers would not be a problem in itself, as it could be in other materials. However, this moisture should be taken into account to subtract it from the water necessary for conforming.

The tests performed on the aforementioned samples can be classified into two sections, physical tests, intended to determine the particle density UNE-EN 1097-7 [83] and laser diffraction granulometry; as well as chemical tests, aimed at determining the different chemical elements in the samples, elemental analysis, loss on ignition and X-ray fluorescence. It is essential to detect those chemical elements that will help the geopolymerization process, as well as those harmful elements that must be monitored in the process.

#### 2.2.2. Conformed of Geopolymers: Physical and Mechanical Tests of the Conformed Samples

Characterized the initial materials and studied their suitability for use in the realization of geopolymers, we proceeded to the conformation of the different families of test samples based on the combination of both industrial by-products, chamotte and biomass bottom ash (BBA).

The starting aluminosilicate, which will be activated later, is the chamotte. Therefore, it is the base element on which it was proceeded to add increasing amounts of the alkaline activator, biomass bottom ash.

This increase was made from 0% to 100%, reflecting all possible combinations of both materials for the further study of the geopolymer conformed. In this way, it is possible to analyze the optimal combination and possible cases in which they reflect the characteristics acceptable by the regulations in this regard. An analytical chemical study of the combination of both elements would be extremely difficult and unrepresentative of reality, since, being industrial by-products, the elements are not high in purity. The different sample groups are represented in Table 1, showing the percentage of each by-product for each group.

It should be noted that samples groups 10C0A and 0C10A, made up of 100% chamotte and 100% biomass bottom ash, respectively, obviously do not produce geopolymers, since there is no activation of aluminosilicates. However, both families have been carried out to physically, mechanically and aesthetically check the variations that occur in the formation of the geopolymer, as well as to be certain that the geopolymer has been formed. From each of the detailed families, six samples were formed in order to have statistically analytical results.

The samples were formed following the same process for all families, this being the one detailed below:

- The chamotte and the biomass bottom ash were mixed until the resulting mass was homogenized and according to the corresponding percentages of each family.
- Subsequently, 20% water was added to the previous mass, mixing again until obtaining the homogenization of the product.
- This resulting mixture was conformed in a steel matrix of internal dimensions of 60 × 30 mm, applying a gradual pressure through a piston until reaching 30 ± 1 MPa. This pressure was maintained for one minute.
- Once the mixture was compacted, the sample was removed, leaving the sample fully conformed.

It should be noted that the percentage of 20% water added to the mixture for conforming was determined empirically to optimize this process. Higher percentages of water caused an excess of water exudation during compression.

Once the samples were made, they were left at room temperature (20 ± 2 °C) for 24 h and at 90 ± 2 °C for another 24 h to remove excess water that has not reacted during the geopolymerization process. As mentioned above, the curing temperature of the geopolymer has a significant influence on the mechanical characteristics. However, if the geopolymer is to replace traditional ceramics, the production times must be similar. Therefore, first a curing at ambient temperature for 24 h is produced to increase the resistance and subsequently, a drying at higher temperature to decrease the production times once the resistance of the geopolymer has been reached. 

Subsequently, and in order for this process to take place in full, once the different samples of the different families were dried, their geometric dimensions were measured and weighed to subsequently undergo a process of continuous recirculation of water (20 ± 2 °C). This process has two main objectives, the first of which is to eliminate possible excess elements that are properly diluted in the water and have not reacted, or have no utility within the geopolymer; on the other hand, to provide the water necessary for the geopolymerization reaction to occur if, at first, it could have been stopped due to a lack thereof. After this continuous water recirculation process, the conformed samples were dried again. Once dried for 24 h at a temperature of 90 ± 2 °C, the geometric dimensions and mass were measured, for the subsequent study of the variation of the physical properties in the geopolymerization process.

Once six samples were obtained for each of the families, the physical properties of the different sample groups were studied through the tests usually used for ceramic materials. These tests were the determination of mass loss, the determination of dimensions UNE-EN 772-16 [84], capillary water absorption UNE-EN 772-11 [85], cold water absorption UNE-EN 772-21 [86], boiling water absorption UNE-EN 772-7 [87], bulk density and open porosity UNE-EN 772-4 [88]. The purpose of carrying out the present tests is the study of the physical characteristics of the materials formed to compare them with traditional ceramics, since the main objective of the project was the replacement of the latter by geopolymers.

Subsequently, the color of the various samples of the families was objectively evaluated. For this, the colorimeter will be used, which will reflect the color of the different samples in combination with the primary colors.

Finally, the mechanical properties of the different families will be studied through the compression test UNE-EN 772-1 [89], that will be able to obtain the resistance of each of them. With this essay, families can be accepted or rejected based on European regulations and their compressive strength.

It should be noted that traditional ceramics formed with red clay for the manufacture of bricks are those that the present work seeks to replace with geopolymers. Therefore, the comparison ceramics were performed with the same forming conditions (water and compaction) and were sintered in the oven at a temperature of 950 ± 10 °C, with heating ramps of 4 °C/min and temperature maintenance for 1 h.

#### 2.2.3. Ageing Tests (Freezing Test) and Fourier Transform Infrared (FTIR) of the Geopolymers

The main purpose of the freezing test was the study of the behavior of the different sample families after the effect produced by a continuous cycle of ice and melt UNE 67028 [90]. In this way, its durability could be evaluated over time and the quality of the geopolymer was obtained before the inclement weather.

There were taken six samples of each family for its development and they were introduced into the melting tank, progressively submerging them at a temperature of 15 ± 2 °C and in a minimum time of 3 h. Subsequently, they were removed and they were left to rest for a period of 1 min, in order to introduce them into a cold room without any contact between them. They were kept in the chamber for 18 h, remaining at least 11 h at the temperature of −15 ± 2 °C. They were subsequently removed from the chamber and introduced into the melting tank for at least 6 h. This process was repeated for a total of 25 cycles.

After performing the 25 test cycles for the samples of the families, the visual inspection was carried out. The objective of the visual inspection testing (VT) is to evaluate the appearance of breaks, spalling and chipping greater than 15 mm, according to the UNE 67028 standard [90]. If any of the defects mentioned in several of the samples of the different families appears, this would be classified as a freezing geopolymer, unsuitable for use.

Sample families that obtained acceptable results in the freezing test were analyzed with Fourier transform infrared. For this purpose, the samples of these families were manufactured again with the process detailed in the methodology. The 10C0A and 0C10A families corresponding to 100% chamotte and 100% biomass bottom ash, respectively, were also analyzed. In this way, it was possible to evaluate the differences that existed between the different spectra of the detailed families and the base materials, thus analyzing the formation of the geopolymer and chemically corroborating its existence.

To carry out this test, the samples were first crushed to a particle size of less than 0.063 mm. The detailed samples were analyzed with the Bruker Tensor20 spectrophotometer (Tensor20, Bruker, Billerica, MA, USA) which allowed the recording of the FTIR spectra of solid, liquid and gaseous samples in the mid and near infrared range. In addition, in this case, it was used in the attenuated total reflectance (ATR). The standard spectral resolution was 4 cm^−1^, with a spectral range of 4000–400 cm^−1^.

## 3. Results and Discussions

The successive sections describe the results of the different tests detailed above in the methodology, including in each of them the partial conclusions that may be derived from their analysis.

### 3.1. Initial Tests of by-Products

This section details the results obtained and the conclusions derived from the tests destined for the determination of the physical and chemical properties of the elements under study, chamotte and biomass bottom ashes.

First of all, and within the physical properties, it has been obtained that the particle density of the chamotte and the bottom ashes of biomass is 2.54 and 2.65 t/m^3^, respectively. Both densities are adequate and similar, so no volume correction would be necessary for the geopolymer conforming process. The results given off are similar to the diversity of materials used in construction, established as the usual particle density 2.65 t/m^3^.

**Figure 1:**
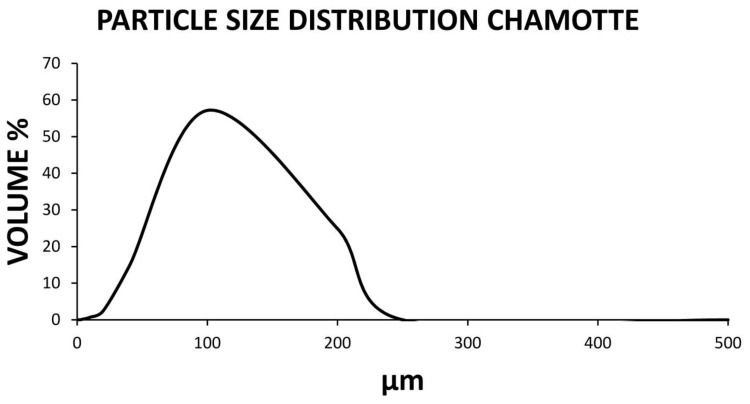
Graph of the laser granulometry of the sieved chamotte by a 0.25 mm sieve.

In turn, Figure 1 shows the particle size distribution of the chamotte (sieved by the 0.25 mm sieve). It is distinguished that the highest percentage of particles have a size between 40 and 200 micrometers. This microscopic granulometry makes chamotte an ideal by-product for use as an aluminosilicate in the conformation of the geopolymers. This fact is due to the fact that its fineness as well as its amorphous form makes an excellent combination with the activator possible.

**Figure 2:**
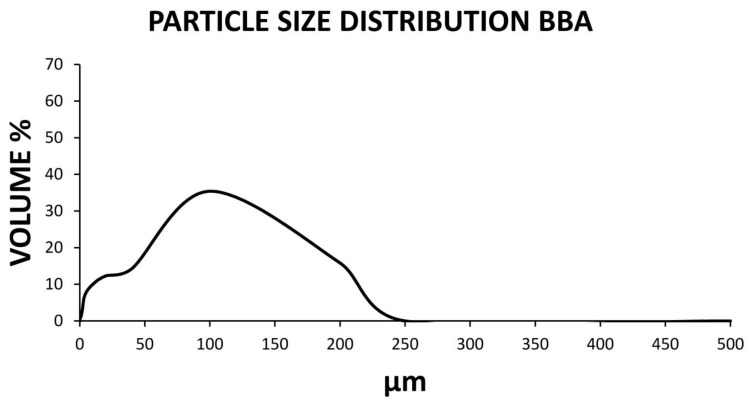
Graph of the laser granulometry of the sieved BBA by a 0.25 mm sieve.

Similarly, Figure 2 shows the particle size distribution of the bottom ash of the biomass sieved by a 0.25 mm sieve after mashing and drying. The distribution of the particles was observed from 10 to 200 um, as a finer material than the chamotte and suitable for use as an activator in geopolymers. It should be noted that different authors have studied and corroborated that the fineness of the materials used in the conformation of the geopolymer greatly influences the final mechanical characteristics.

Once the physical properties were evaluated and the results obtained for the conformation of geopolymers were acceptable, the chemical properties were studied. The first test performed was that of elementary analysis, to determine the percentage of carbon, nitrogen, hydrogen and sulfur present in both samples.

The results of the elemental analysis test for the chamotte and the biomass bottom ash, observable in Table 2, reflect a very low percentage of carbon. This fact is obvious, since they derive from sintering or combustion procedures that are carried out at very high temperatures. On the other hand, it should be highlighted that the value of sulfur in both samples was zero—without assuming a problem to be analyzed later. If, on the contrary, there was sulfur in one of the two samples, it should be studied later to prevent environmental pollution problems.

This test was complemented with that of loss on ignition, which is detailed in Table 3 for both materials.

As the results reflect, the loss on ignition in both samples was markedly reduced. This fact, as mentioned in the previous trial, is due to the production process of both by-products, which was produced at high temperatures. The loss on ignition of the chamotte is lower than that of the BBA, since, for its production through ceramic materials, a sintering temperature of the clay material is necessary. In the case of BBA, even when high temperatures are reached, the process is much faster so it can lead to unburning.

The X-ray fluorescence of chamotte is shown in Table 4 and reflects an elemental composition similar to that of any traditional ceramic. The silicon-aluminum ratio is suitable for the formation of geopolymers and in turn, the percentages of magnesium, calcium and iron are just right so as not to cause any problems. Therefore, it can be concluded that chamotte is a good source of aluminosilicate for the manufacture of geopolymers, without contaminating or containing harmful elements that could interfere with the process.

On the other hand, the X-ray fluorescence of biomass bottom ash, shown in Table 5, reflects a high percentage of potassium. This fact is very interesting and necessary for its use as an activator of the mentioned aluminosilicate. The other two majority elements are silicon and calcium which appear to a lesser extent and do not represent a problem for the geopolymer, as they even increase the silicon–aluminum ratio and can help to obtain resistance. The rest of the elements present in the biomass bottom ash sample do not represent a problem for the viability of the conformed geopolymer and there are no elements hazardous to the environment. However, they are a burden on the material unlike the use of pure potassium hydroxide, which is remedied for its lower price and a higher percentage of addition.

### 3.2. Physical and Mechanical Tests of the Conformed Samples

**Figure 3:**
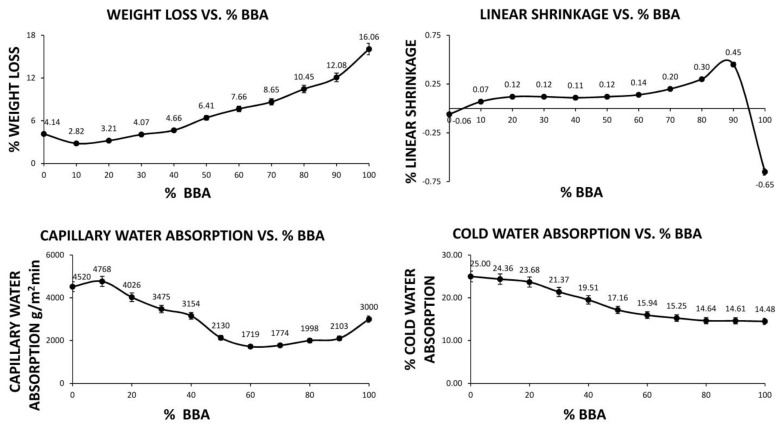
Graphs of the loss of weight, linear shrinkage, capillary water absorption and the cold water absorption of the different sample groups of geopolymers.

The families made of samples with the different percentages of added chamotte and BBA were tested to study their viability. Figure 3 shows the results of the loss of weight, linear shrinkage, capillary water absorption and cold water absorption of the different samples of the families after the water recirculation process. That is, after conforming and subsequent drying process in an oven, the dry weight was determined and subsequently, they were submerged in a recirculated bath and dried after 24 h.

As can be seen in Figure 3, the percentage of weight loss is increasing with respect to the percentage of BBA about mixing in the geopolymer. This fact is mainly based on the ability of the geopolymerization process in the circulation of water to eliminate those superfluous elements that are not part of the geopolymer’s structure. These elements are mostly present in the BBA, since the chamotte is a stable material because of its sintering process. Compared to a traditional ceramic after sintering, which has a weight loss of around 9.5%, it can be deduced that it is quite similar, in most cases being even lower.

The linear shrinkage values for the 100% chamotte and 100% BBA families are substantially different from the other families. This is because there is no combination of the two by-products and the geopolymer cannot be formed. On the other hand, the families formulated with a combination of both by-products have a greater linear to medium shrinkage that increases the percentage of BBA, which is not excessively high in any case. This fact is corroborated when comparing the linear shrinkages obtained with a traditional ceramic. A red clay ceramic after sintering has an average linear shrinkage of 2.7%.

As in the previous case, the water absorption rate of the families with 100% chamotte and 100% BBA is significantly different from the rest of the families. In the families composed of both wastes, a tendency towards a decrease in the absorption rate is observed with an increase in the addition of BBA. This fact implies the creation of a denser material and with less open porosity with the increase in the percentage of BBA. The reduction in the water absorption rate creates a material suitable for outdoor use, since contact with water would not cause great absorption and an increase in the weight of the material to be supported by the structure. In comparison, a traditional ceramic has a capillary water absorption of 1700 g/m^2^·min, similar to the samples formed with 60% BBA and higher percentages. 

As with the capillary water absorption, rate cold water absorption reflects a reduction in the absorption capacity of samples with a higher percentage of BBA in their formulation. This fact predicts a higher density of the materials created and a lower porosity, which in turn could lead to higher compressive strength. The traditional ceramics usually have a cold water absorption of 13%, something lower than the values obtained and derived from the sintering and conforming process.

**Figure 4:**
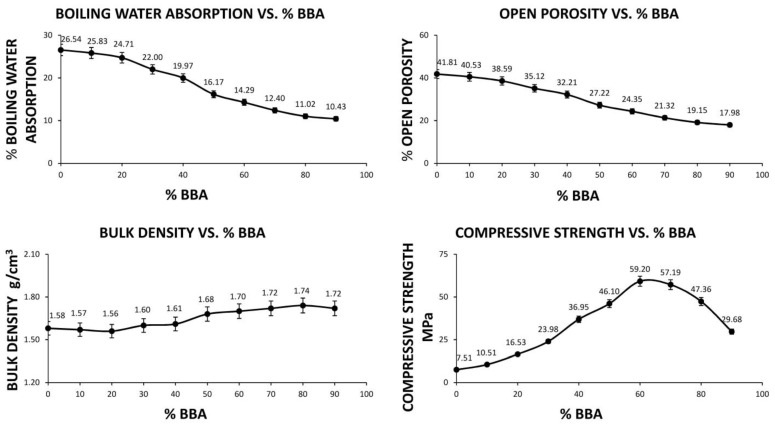
Graphs of boiling water absorption, open porosity, bulk density and compressive strength of the different samples groups of geopolymers.

At the same time, the results of the boiling water absorption, open porosity, bulk density and compressive strength tests for the different sample groups are detailed in Figure 4. The progressive addition of BBA causes a lower boiling water absorption, a higher bulk density of the test samples and a lower open porosity of the families. This fact will be directly related to the quality of the geopolymer.

It should be noted that the samples made of 100% BBA cracked and collapsed during the boiling water absorption test, therefore they were totally discarded from further interpretation. The unsuitability of this family does not suppose a problem, since it cannot be considered a geopolymer as it is only made up of BBA and there is no suitable aluminosilicate. In family 10C0A, a similar fact occurs, only the aluminosilicate exists and not the activator so it cannot be considered a geopolymer. However, both families show that the geopolymer has been formed as there are great differences in its physical and mechanical properties.

The boiling water absorption and the open porosity in a traditional ceramic is usually 12% and 24%, respectively. As can be seen in Figure 4, the families with percentages of 60% BBA addition in the mixture coincide approximately. On the other hand, lower values are obtained for higher percentages of addition of BBA at 60%.

On the other hand, a standard ceramic usually has a bulk density of around 2 t/m^3^, while geopolymers in all families have lower densities. This decrease in density, far from being a problem, can become a strength, because if the adequate resistances prescribed by the regulations are achieved, having a lower density makes possible a lower thermal insulation, as well as a better acoustic insulation. On the other hand, its lower weight makes it possible to create lighter claddings that do not overload the structure of the building.

Finally, the compression strength test of the conformed samples of the different families of geopolymers is essential to evaluate the formation of the geopolymer. Moreover, with this test, the appropriate percentage of the combination of chamotte and BBA will be selected for production. 

Firstly, the results clearly reflect the formation of a geopolymer structure, since it can be seen that the compressive strength of the samples with a combination of chamotte and BBA increases with respect to the samples conformed with chamotte alone. In turn, over 60% of BBA on the mix creates a material which, although it has a higher density and lower porosity, has a reduction in compressive strength. This is mainly due to the fact that there is no adequate proportion of chamotte and BBA, so that only a percentage of the BBA reacts with the chamotte and all the unmixed BBA remains in excess. This fact decreases the resistance notably as it is an inert load. It should be noted that in this research the material is composed entirely of waste, so that in these there are a number of chemical compounds that do not only not favor the geopolymerization reaction but lead to a decrease in resistance. 

Both conclusions confirm the geopolymer structure formed, with maximum compressive strength around the combination of 60% BBA and 40% chamotte. However, there is a range of the combination of both by-products that comply with the regulations on the resistance of ceramic materials, more specifically beginning with family 9C1A. This European standard sets a minimum compression strength for red clay bricks of 10 MPa.

The ageing test will rule out those families of samples that do not have adequate behavior in service, even though they reflect adequate initial mechanical characteristics. In turn, the colorimetric test will classify the samples according to color, which is essential within ceramic materials. The ultimate aim is to create a resistant but pleasantly aesthetic material that is accepted by the market. 

**Figure 5:**
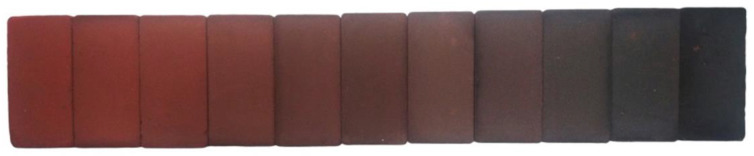
Image of the different families of samples from family 10C0A (left) to family 0C10A (right).

Figure 5 shows an orderly representation of a sample from each of the families of geopolymers. As can be seen, there is a darkening of the samples due to the increasing percentage of BBA. At the ends are the colors of the chamotte and the BBA, respectively. In the central zone are the intermediate combinations of both residues. Since the aesthetics of an element are a personal appreciation, and ultimately it is the market that must choose it, it can only be identified faithfully in order to establish a color scale that does not vary according to the photograph taken.

In order to determine the color of each sample accurately, the colorimeter was used, giving the following values for the primary colors red, green and blue, as detailed in Table 6.

The color coordinates of the different groups of samples are another characteristic of the material, not limited by the regulations but by the quality controls of the industry. It is usual that a ceramic material incorporating waste is not accepted by the industry because of the color it reflects, even if it has adequate physical and mechanical characteristics. The quality criteria established by the producing companies have maximum permissible variations in the color of the final material, and therefore the addition of waste that varies sharply in color is rejected. In this case, it can be seen that the variation in color is gradual and towards darker shades, which is important and easy to market.

### 3.3. Ageing Tests (Freezing Test) and Fourier Transform Infrared (FTIR) of the Geopolymers

Once all the parameters of the different families, physical, mechanical and aesthetic, had been determined, the freezing test was carried out. 

The ultimate aim of this test was to study the behavior of the different families in service, i.e., to study the variation in the initial characteristics over time. To evaluate ageing, this test requires a visual inspection after 25 freezing and defrosting cycles, determining which families are affected and should be discarded. The affected geopolymers will be called freeze geopolymers and will be rejected.

**Figure 6:**
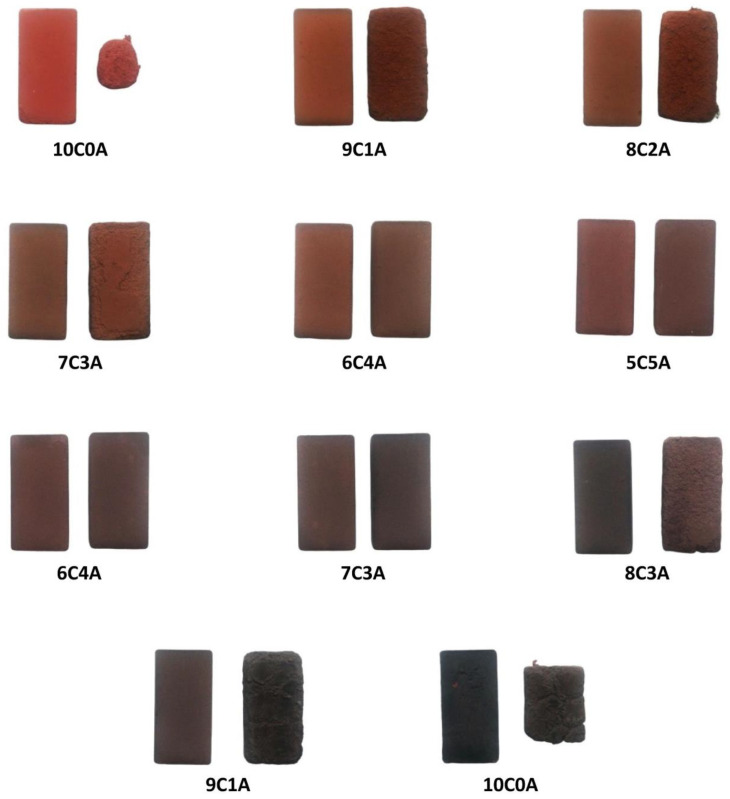
Image of the samples before the freezing test (left) and after the freezing test (right) for each of the geopolymer families studied.

Figure 6 shows the picture of the different sample families before and after the freezing test. For comparison, one sample is taken that has been tested and another that has not been tested for each family.

After performing the freezing test and the observation of the above pictures, it can be concluded that only sample families 6C4A, 5C5A, 4C6A and 3C7A are suitable. Families 10C0A, 9C1A, 8C2A, 7C3A, 2C8A, 1C9A and 0C10A are freezing geopolymers, as they present spalling and flaking of more than 15 mm. Freezing geopolymers are rejected because they may not represent a sufficient quality of service. 

On the basis of this result, it can be commented that although the physical and mechanical characteristics of the previous families, except for families 10C0A and 0C10A, were within the current regulations, the freezing test revealed a subgroup of samples with better mechanical and physical behavior during their useful life. These families will definitely be the ones considered as possible solutions, corresponding to combination percentages of 40% BBA with 60% chamotte up to 70% BBA with 30% chamotte.

The acceptable sample families listed above were then subjected to Fourier transform infrared (FTIR) analysis. For this purpose, new samples were made with the process detailed in the methodology and analyzed in order to compare the spectra. In turn, the families 10C0A and 0C10A corresponding to 100% chamotte and 100% BBA, respectively, were analyzed. In this way, it is easy to observe in the comparison of the spectra the differences that exist between them as well as the modifications that are produced by the process of geopolymerization.

**Figure 7:**
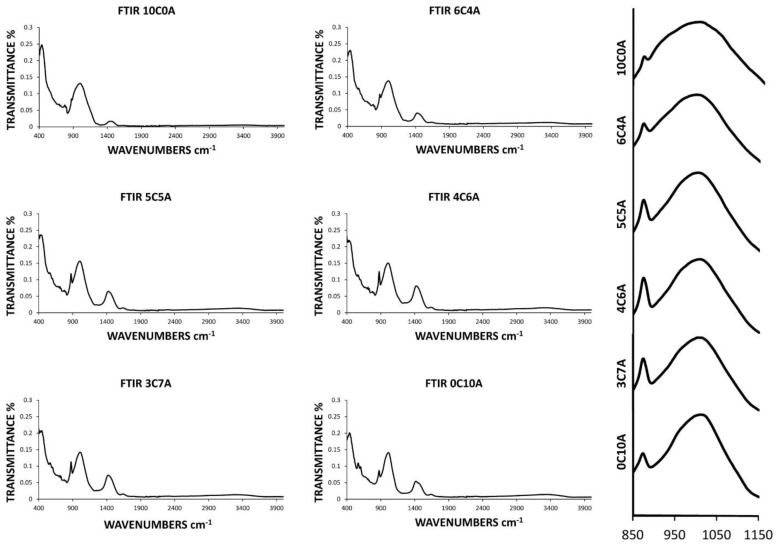
Fourier transform infrared (FTIR) analysis of the families with acceptable results (6C4A, 5C5A, 4C6A and 3C7A), as well as of the family with 100% chamotte (10C0A) and the family with 100% BBA (0C10A). On the right, a comparison of the intensity of the spectra for the families detailed in the region 850–1150 cm^−1^ is shown.

Figure 7 shows the spectra of all the detailed families as well as in the right margin the amplification of all the spectra between 850 and 1150 cm^−1^. It can be seen how due to the geopolymerization process, the asymmetric stretching frequency changes to a lower value for the families 6C4A, 5C5A, 4C6A and 3C7A than the one of the band presenting the chamotte and the BBA around 1010 cm^−1^. This is because AlO4 partially replaces SiO_4_ and changes the chemical environment of the Si–O bond. On the other hand, the comparison of the spectra in the 850–1150 cm^−1^ zone shows that the intensity of the frequency band detailed above and that of 875 cm^−1^ increases for the 6C4A, 5C5A, 4C6A and 3C7A geopolymer families with respect to the chamotte and BBA bands. The increase in intensity indicates an increase in chain length and more aluminosilicate gel formed, i.e., a more complete geopolymerization process. It should be noted that the higher intensity is reflected in the 4C6A group of samples, which in turn has been the most resistant family. It can be concluded that the Fourier transform infrared (FTIR) analysis coincides with the results with the previous compression test.

## 4. Conclusions

The development of the research methodology present in this work has led to a series of conclusions derived from each test. In order to obtain an objective and representative final conclusion of this investigation, the conclusions derived from each of the sub-sections of the methodology are set out below:

- The physical–chemical characterization of the chamotte and the biomass bottom ashes showed the suitability of both materials for the conformation of geopolymers. The elemental composition of the chamotte provides the perfect base of aluminosilicate, in combination with the high percentage of potassium present in the biomass bottom ashes. On the other hand, the similarity between the densities of both by-products and their microscopic granulometry facilitates the mixing process.
- The physical tests carried out on the families of samples conformed have reflected logical and statistically representative behavior. The loss of weight and linear shrinkage increased as the percentage of BBA in the mix increased. However, the bulk density is much lower than that of a traditional ceramic, which is of interest for other properties such as thermal or acoustic insulation. On the other hand, the rate of capillary water absorption, the cold water absorption and boiling water absorption, as well as the open porosity, decreased as the percentage of BBA in the mixture increased.
- The mechanical tests reflected a perfect quadratic curve, with a maximum of around 60% biomass bottom ashes in the mixture. However, all the families, except for 10C0A and 0C10A, showed adequate resistance behavior according to the regulations in force.
- The freezing tests determined that only the 6C4A, 5C5A, 4C6A and 3C7A families have adequate resistance to the ageing test.
- The Fourier transform infrared (FTIR) analysis reflected the formation of the geopolymer for the 6C4A, 5C5A, 4C6A and 3C7A sample groups.
- Geopolymers with acceptable results are formed with 40% BBA and 60% chamotte up to 70% BBA and 30% chamotte, the optimum combination being 60% BBA and 40% chamotte.

On the basis of the partial conclusions mentioned above and obtained from the detailed investigation methodology, it can be concluded that it is possible to produce geopolymers with physical, mechanical and aesthetic characteristics similar to those of traditional ceramics. Therefore, a sustainable material can be developed, thanks to the use of industrial by-products and to the low gas emissions of the manufacturing process, with appropriate characteristics and composed entirely of waste.

## Figures and Tables

**Table 1:** Sample groups composed of geopolymers with different combination percentages of chamotte and biomass bottom ash (BBA).

Samples Groups	Chamotte, %	BBA, %
10C0A	100	0
9C1A	90	10
8C2A	80	20
7C3A	70	30
6C4A	60	40
5C5A	50	50
4C6A	40	60
3C7A	30	70
2C8A	20	80
1C9A	10	90
0C10A	0	100

**Table 2:** Elemental analysis of the chamotte and the BBA.

Sample	Nitrogen, %	Carbon, %	Hydrogen, %	Sulfur, %
Chamotte	0.00 ± 0.00	0.24 ± 0.01	0.08 ± 0.00	0.00 ± 0.00
BBA	0.05 ± 0.00	4.64 ± 0.14	0.48 ± 0.02	0.00 ± 0.00

**Table 3:** Loss on ignition for the chamotte and the BBA.

Sample	Loss on Ignition, %
Chamotte	1.74 ± 0.10
BBA	8.16 ± 0.19

**Table 4:** X-ray fluorescence of the chamotte.

Element	wt, %
Si	27.32 ± 0.12
Al	8.16 ± 0.10
Ca	5.95 ± 0.10
Fe	4.57 ± 0.09
K	3.80 ± 0.09
Mg	1.92 ± 0.05
Ti	0.455 ± 0.023
Sx	0.119 ± 0.006
Na	0.201 ± 0.012
P	0.0965 ± 0.0048
Mn	0.0665 ± 0.0033
Sr	0.0523 ± 0.0030
Zr	0.0375 ± 0.0037
V	0.0209 ± 0.0018
Ni	0.0242 ± 0.0016
Rb	0.0208 ± 0.0043
Cr	0.0146 ± 0.0017
Pt	0.0162 ± 0.0039
Cl	0.0107 ± 0.0008
Ru	0.0070 ± 0.0026
Total weight % oxygen	45.39 ± 0.47

**Table 5:** X-ray fluorescence of the biomass bottom ash.

Element	wt, %
K	23.91 ± 0.19
Si	11.21 ± 0.10
Ca	11.10 ± 0.13
Px	3.58 ± 0.06
Mg	4.21 ± 0.08
Al	2.57 ± 0.06
Fe	1.33 ± 0.05
Sx	0.230 ± 0.011
Na	0.229 ± 0.019
Cl	0.255 ± 0.013
Ti	0.128 ± 0.006
Sr	0.0859 ± 0.0043
Mn	0.0442 ± 0.0022
Cu	0.0240 ± 0.0016
Ni	0.0221 ± 0.0012
Cr	0.0135 ± 0.0013
Zr	0.0106 ± 0.0027
Rb	0.0070 ± 0.0035
Zn	0.0047 ± 0.0016
V	0.0024 ± 0.0012
Total weight % oxygen	32.89 ± 0.36

**Table 6:** RGB color coordinates of the samples conformed by the chamotte and the BBA of the different groups.

Groups	Chamotte, %	BBA, %	Red	Green	Blue
10C0A	100	0	379 ± 19	182 ± 9	115 ± 7
9C1A	90	10	249 ± 12	119 ± 5	77 ± 3
8C2A	80	20	253 ± 8	126 ± 7	84 ± 4
7C3A	70	30	232 ± 10	126 ± 7	87 ± 4
6C4A	60	40	192 ± 10	109 ± 5	78 ± 3
5C5A	50	50	177 ± 10	107 ± 6	79 ± 3
4C6A	40	60	170 ± 6	112 ± 4	85 ± 3
3C7A	30	70	155 ± 8	115 ± 4	93 ± 3
2C8A	20	80	147 ± 9	115 ± 6	97 ± 5
1C9A	10	90	142 ± 8	122 ± 6	109 ± 5
0C10A	0	100	118 ± 5	115 ± 6	110 ± 5

## Data Availability

Data is contained within the article.
